# Variation of vital signs with potential to influence the performance of qSOFA scoring in the Ethiopian general population at different altitudes of residency: A multisite cross-sectional study

**DOI:** 10.1371/journal.pone.0245496

**Published:** 2021-02-04

**Authors:** Jonas Früh, Andre Fuchs, Tafese Beyene Tufa, Loraine Früh, Zewdu Hurissa, Hans Martin Orth, Johannes Georg Bode, Kirsten Alexandra Eberhardt, Dieter Häussinger, Torsten Feldt

**Affiliations:** 1 Hirsch Institute of Tropical Medicine, Asella, Ethiopia; 2 Department of Gastroenterology, Hepatology and Infectious Diseases, University Hospital, Heinrich Heine University, Düsseldorf, Germany; 3 College of Health Sciences, Arsi University, Asella, Ethiopia; 4 Bernhard Nocht Institute for Tropical Medicine, I. Department of Medicine, University Medical Center Hamburg-Eppendorf, Hamburg, Germany; Vanderbilt University Medical Center, UNITED STATES

## Abstract

**Introduction:**

The physiological range of different vital signs is dependent on various environmental and individual factors. There is a strong interdependent relationship between vital signs and health conditions. Deviations of the physiological range are commonly used for risk assessment in clinical scores, e.g. respiratory rate (RR) and systolic blood pressure (BP_sys_) in patients with infections within the quick sequential organ failure assessment (qSOFA) score. A limited number of studies have evaluated the performance of such scores in resource-limited health care settings, showing inconsistent results with mostly poor discriminative power. Divergent standard values of vital parameters in different populations, e.g. could influence the accuracy of various clinical scores.

**Methods:**

This multisite cross-sectional observational study was performed among Ethiopians residing at various altitudes in the cities of Asella (2400m above sea level (a.s.l.)), Adama (1600m a.s.l.), and Semara (400m a.s.l.). Volunteers from the local general population were asked to complete a brief questionnaire and have vital signs measured. Individuals reporting acute or chronic illness were excluded.

**Results:**

A positive qSOFA score (i.e. ≥2), indicating severe illness in patients with infection, was common among the studied population (n = 612). The proportion of participants with a positive qSOFA score was significantly higher in Asella (28.1%; 55/196), compared with Adama, (8.3%; 19/230; p<0.001) and Semara (15.1%; 28/186; p = 0.005). Concerning the parameters comprised in qSOFA, the thresholds for RR (≥22/min) were reached in 60.7%, 34.8%, and 38.2%, and for BP_sys_ (≤100 mmHg) in 48.5%, 27.8%, and 36.0% in participants from Asella, Adama, and Semara, respectively.

**Discussion:**

The high positivity rate of qSOFA score in the studied population without signs of acute infection may be explained by variations of the physiological range of different vital signs, possibly related to the altitude of residence. Adaptation of existing scores using local standard values could be helpful for reliable risk assessment.

## Introduction

The normal range of vital signs depends on various environmental and individual factors, and there is a strong interdependent relationship between vital signs and health condition [1, 2]. Deviations of physiological parameters, including respiratory rate (RR) and blood pressure (BP), from the normal range are used in several clinical scores, e.g. the qSOFA (quick sequential organ failure assessment) score. The qSOFA score has been developed as a tool for the identification of patients who are at greater risk for a poor outcome among patients with suspected infection outside the intensive care unit (ICU) [3]. It uses three criteria, assigning one point for low systolic blood pressure (BP_sys_ ≤100 mmHg), high respiratory rate (≥22 breaths per min), or altered mentation (Glasgow coma scale (GCS) <15). Thus, the score ranges from 0 to 3 points and patients with 2 or more qSOFA points are likely to be septic and are at high risk for an unfavorable outcome [3]. Key advantages of the qSOFA are the easy and universal availability of the comprised parameters in clinical settings. A limited number of studies evaluating the performance of sepsis scores in low-resource health care settings have shown inconsistent results with mostly poor discriminative power and high variability across different study sites and settings [4–10]. Previous studies conducted elsewhere have mostly found lower BP, higher resting heart rate (HR) and lower peripheral oxygen saturation (SpO_2_) in people living at high altitudes compared to lowlanders [11–13], although some studies have also documented a rise in BP with increasing altitude [14, 15].

Divergent physiological ranges of the applied vital signs in different populations could be a reason for heterogeneity of the performance of vital sign-dependent clinical scores as the qSOFA score in different populations. A possible cause of such heterogeneities may be attributed to adaptation mechanisms of the local population to higher altitudes. Thus, we investigated the potential influence of variations within the physiological range of vital signs in the general healthy population residing at different altitudes in Ethiopia on the performance of the qSOFA score.

## Materials and methods

The study has been approved by the appropriate Ethical Review Committee (ERC) of the College of Health Sciences, Arsi University, Asella, Ethiopia (project number: A/CHS/RC/72/18). All volunteer healthy participants gave verbal consent after study procedures were thoroughly explained in local language by the study team before data acquisition. The method of verbal informed consent was approved by the Ethical Review Board, considering the high rate of illiteracy and that no invasive procedures were performed. The data were analyzed anonymously. For this multisite cross-sectional observational study, we selected three study sites in Ethiopian cities at different altitudes. Asella was the study site located at the highest altitude (2400 m a.s.l.). Second, we aimed for a study site at the lowest possible altitude within the country, inhabited by a population with similar descent. Since Ethiopia is a landlocked country with large shares of highlands and the areas with the lowest altitude in the country are barren, hostile deserts where hardly any people live, no inhabited area at sea level was available. Therefore, Semara, as one of the cities of the country located at the lowest altitude (400 m a.s.l.), was selected as study site. Third, for comparison at mid-level altitude, the city of Adama (1620 m a.s.l.) was chosen. In order to investigate comparable populations at the respective study centers and therefore avoid selection errors, we selected urban centers at the respective altitudes as study sites. Reliable data on socioeconomic differences between the study sites were not available.

**Fig 1:**
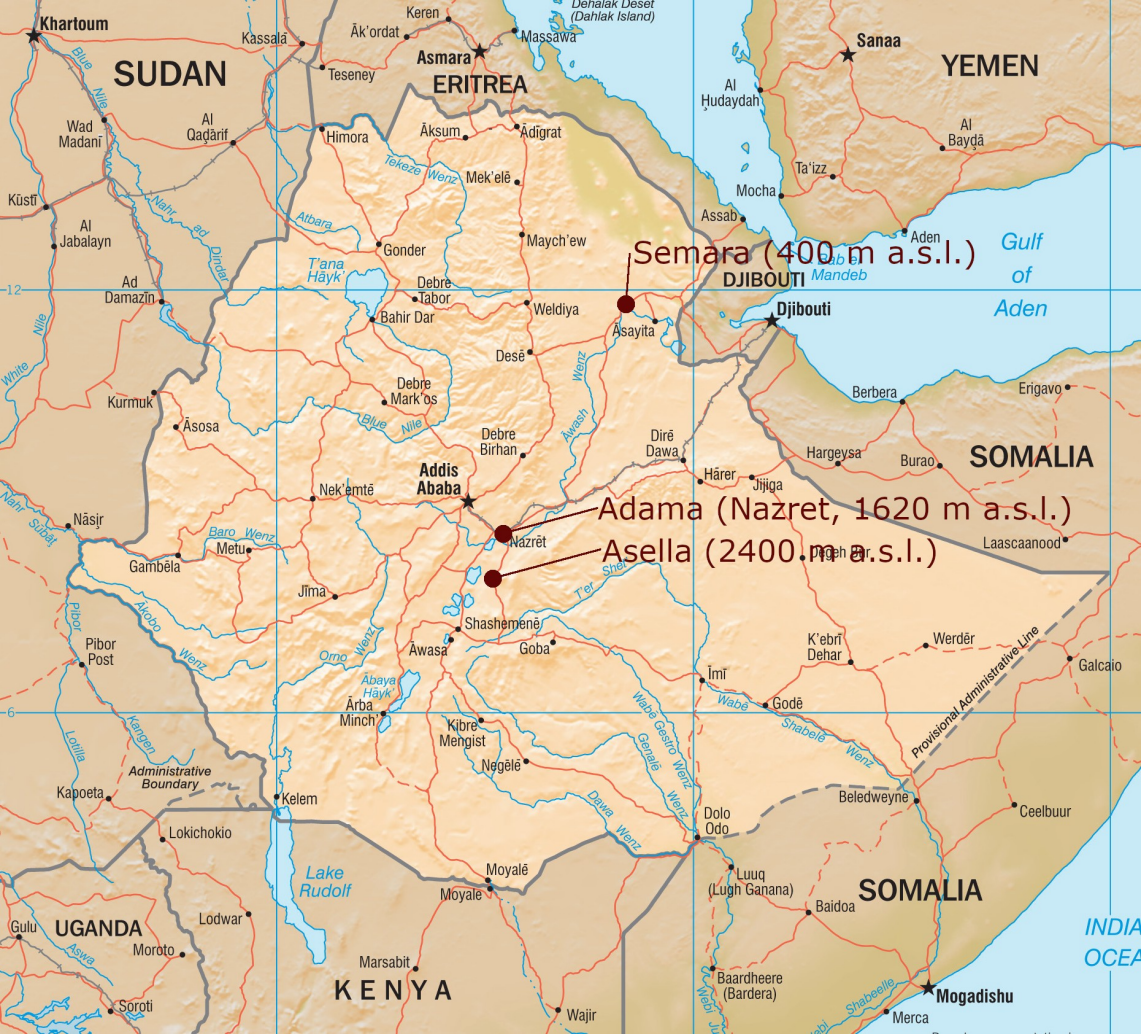
Geographical position of the three study sites in Ethiopia. The original map was downloaded from https://www.cia.gov/static/c383432a1174420f80c37d230bdfc5ee/Ethiopia_Physiography.jpg and modified.

The study was conducted between December 2018 and March 2019 among adult alert volunteers with a minimum age of 16 years (Fig 1). The selection of volunteers was randomly made among pedestrians on the street at each study site. Participants were included for measurements during daytime for two subsequent days in order to achieve a sufficiently large sample size. A mobile medical unit was set up in a tent in a busy area downtown at each of the study sites and random passers-by were invited to have their vital signs checked and to participate in our investigation. Apart from the age limit and the residency in the assigned area, no other initial study eligibility criterion was applied. This approach of random selection of subjects in a busy area of the respective city, populated by local people who pursue their ordinary activities was chosen in order to achieve inclusion of a representative cross-section of the local population. The physiological parameters like body temperature (T), BP_sys_, RR, SpO_2_ and HR were measured non-invasively with medical infrared thermometers, photo optic finger clip pulse oximeters, and aneroid sphygmomanometers. Height and weight were measured from which body mass index (BMI) was calculated by two trained nurses. Prior to these measurements, the volunteers were asked to come to rest in a designated waiting area for not less than 5 minutes, which is in conformity with official recommendations and international guidelines [16, 17]. To avoid measurement errors in pulse oximetry, the measurement was performed only on clean fingers without nail polish. The participants were asked to rest their arm during the measurement. In addition, data on socio-demographic background, current health condition, as well as chronic diseases were collected using a standardized questionnaire. In order to rule out potential influences on vital parameters by medical conditions, participants with acute or previously known chronic illnesses were excluded *a posteriori*. The qSOFA was calculated using the cutoffs of ≥22/min for RR, and ≤100 mmHg for (BP_sys_). Since all participants were fully conscious and responsive during the study procedures, the GCS, assessing mental alteration, was graded unimpaired (15 points) in all participants. Continuous variables were expressed as median (interquartile range, IQR). Post-hoc sample size calculation and power analysis were performed using the approach by Cohen, J. and the R-Package “pwr” [18]. Multigroup comparisons were done using the Kruskal-Wallis Test or one-way ANOVA. Additionally, pairwise comparisons between group levels at the different study sites were performed and adjusted for multiple comparisons using the false discovery rate approach. Categorical variables were compared using either the χ2 test or the Fisher exact test, as appropriate. To evaluate the association of the place of residency with the qSOFA score, a multiple ordinal regression model was used and adjusted for other covariates. An alpha of 0.05 was determined as the cutoff for significance. All statistical analyses were performed using R (version 3.6.3, R Foundation for Statistical Computing, Vienna, Austria).

## Results

**Fig 2:**
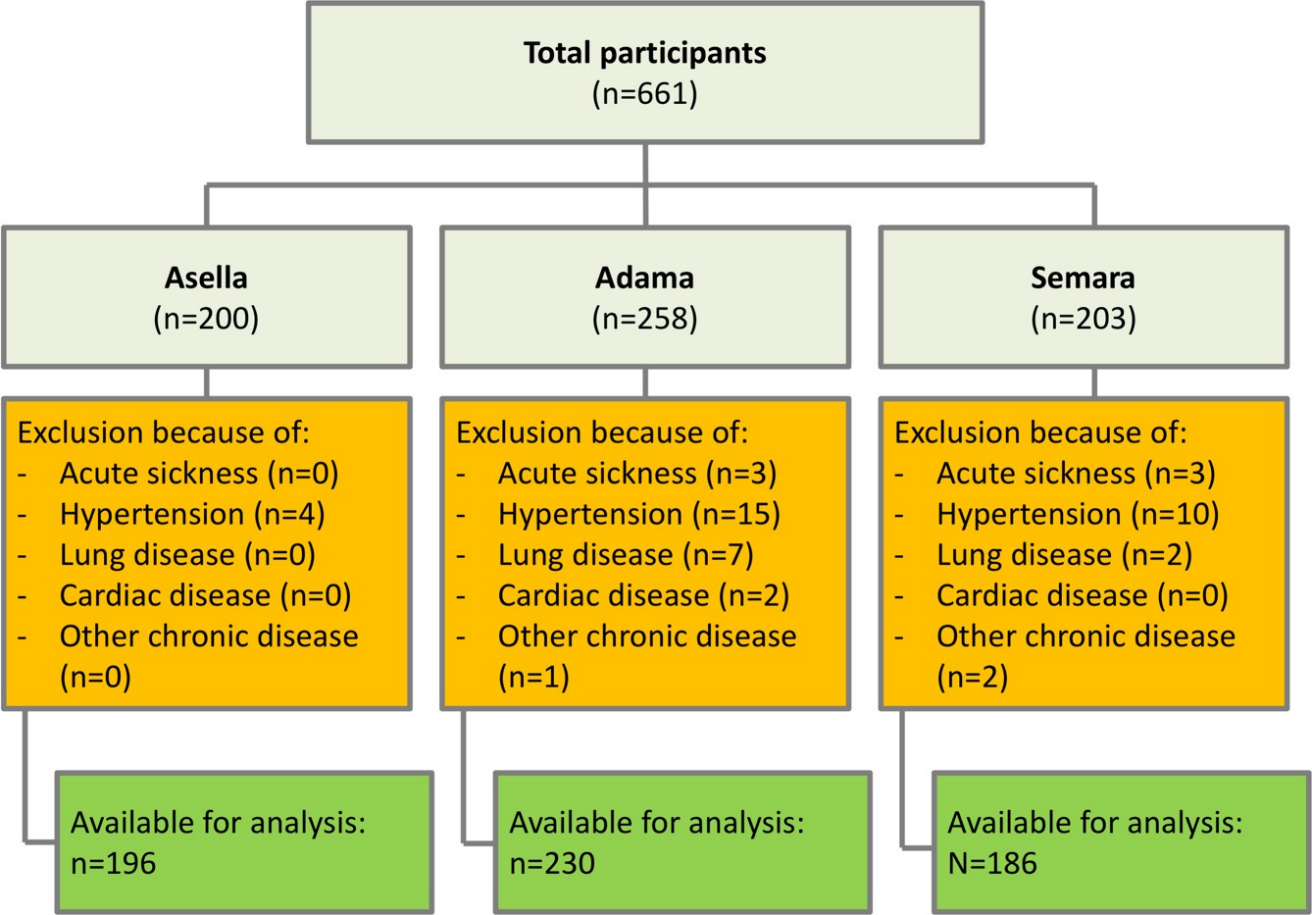
Flow chart of inclusions and exclusions at the different study sites. A total of 661 participants were recruited. After screening for any sickness or disease, the remaining participants used for the analysis was 612.

A total of 612 participants were included in the final analysis. Forty-nine Participants (7.4% of the original study collective) were excluded prior to the final data analysis due to an acute (0.9%) or previously known chronic illnesses (6.5%), primarily arterial hypertension and lung diseases, including tuberculosis (see Fig 2). The mean age of included participants was 31.5 ±12.8 years and 30.6% of them were female (Table 1). All participants were fully alert and oriented and GCS was graded 15/15.

**Table 1:** Demographic parameters.

		Total (n = 612)	Asella (n = 196)	Adama (n = 230)	Semara (n = 186)	p-value (group differences)
**Age in years**	Mean ± SD	31.5 ± 12.7	26.7 ± 10.6	36.0 ± 13.4	31.1 ± 12.1	<0.001
**Sex**	male, n (%)	425 (69.4)	125 (63.8)	148 (64.3)	152 (81.7)	<0.001
	female, n (%)	187 (30.6)	71 (36.2)	82 (35.7)	34 (18.3)	
**Ethnicity**	Oromo, n (%)	293 (47.9)	155 (79.1)	132 (57.4)	6 (3.2)	<0.001
	Amhara, n (%)	179 (29.2)	21 (10.7)	52 (22.6)	106 (57.0)	
	Afar, n (%)	58 (9.5)	0 (0)	0 (0)	58 (31.2)	
	Gurage, n (%)	24 (3.9)	7 (3.6)	17 (7.4)	0 (0)	
	Tigray, n (%)	14 (2.3)	3 (1.5)	8 (3.5)	3 (1.6)	
	Wolayita, n (%)	8 (1.3)	0 (0)	2 (0.9)	6 (3.2)	
	Silete, n (%)	7 (1.1)	4 (2.0)	3 (1.3)	0 (0)	
	Somali, n (%)	1 (0.2)	0 (0)	0 (0)	1 (0.5)	
	Other, n (%)	17 (2.8)	2 (1.0)	10 (4.3)	(5 (2.7)	
	not stated, n (%)	11 (1.8)	4 (2.0)	(2.6)	1 (0.5)	

**Fig 3:**
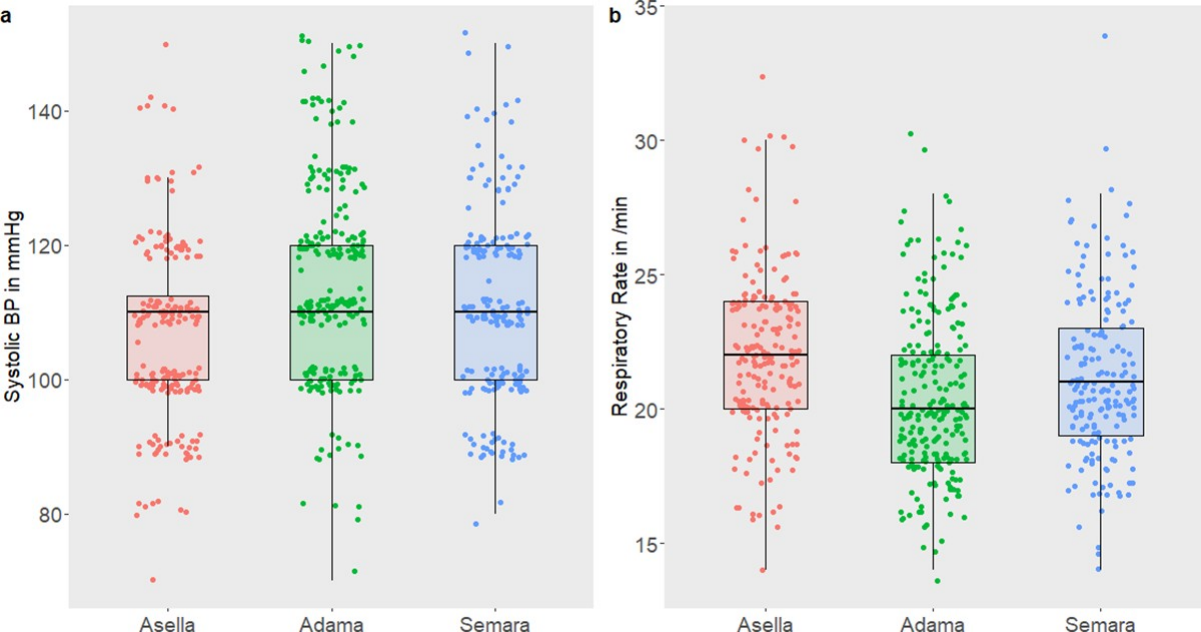
Scatter plots of the vital parameters systolic blood pressure in mmHg (a) and respiratory rate in breaths/min (b) according to areas of residency. Individual measurements of participants are represented by colored dots, and medians as horizontal lines of box plots.

**Fig 4:**
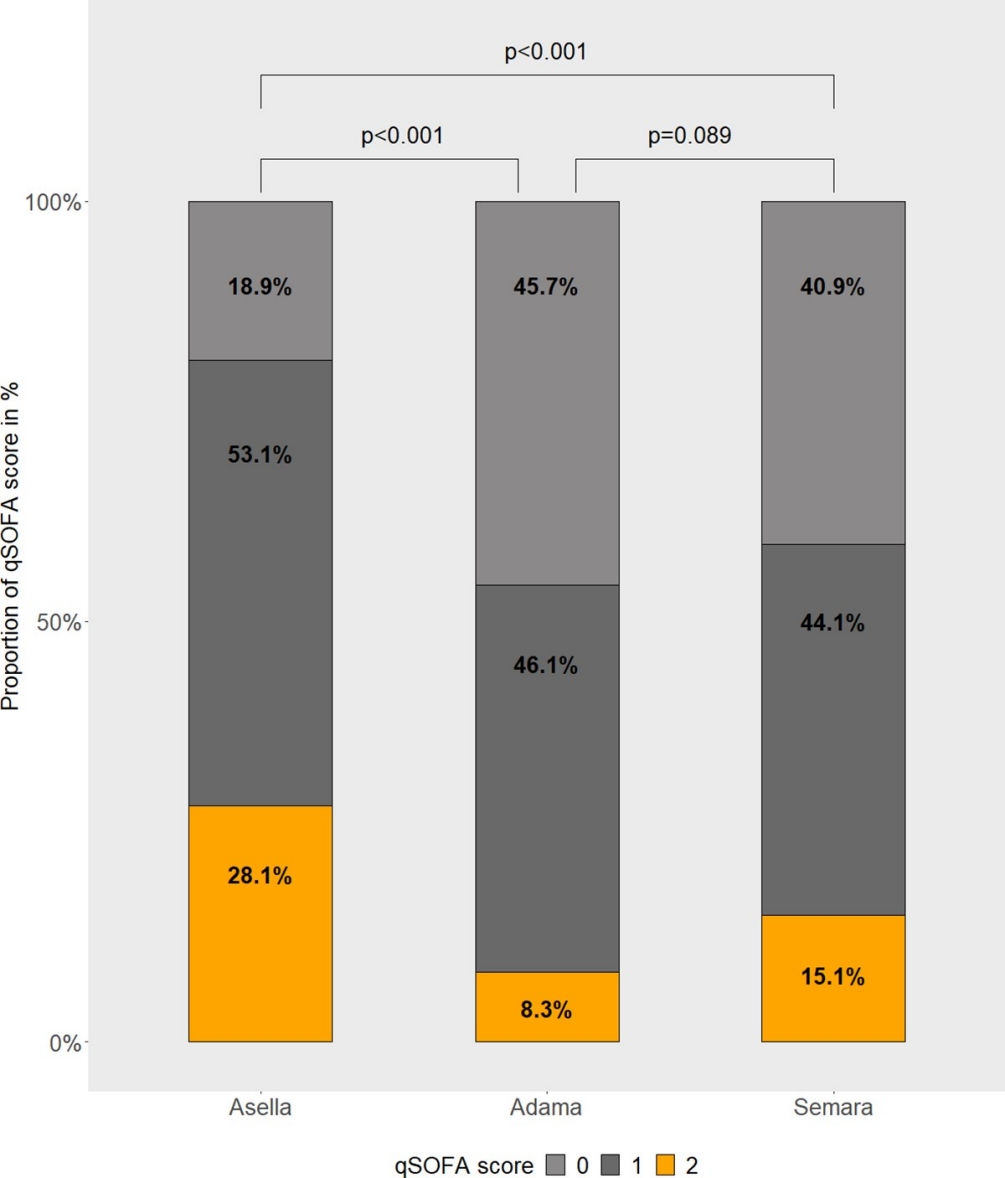
Proportions of qSOFA categories according to study site.

For details on vital parameters see Table 2 and Fig 3. Interestingly, the majority of vital parameters differed significantly across the sites (Table 2). The median respiratory rate was significantly higher in Asella compared with the other sites (22 [IQR 20–24], 20 [IQR 18–22], and 21 [IQR 19–23] /min in Asella, Adama, and Semara, respectively), whereas the median systolic blood pressure was lower in participants from Asella (110 [IQR 100–113], 110 [IQR 100–120], and 110 [IQR 100–120] mmHg in Asella, Adama, and Semara respectively). With regard to the qSOFA, RR threshold was reached in 60.7% (77/196), 34.8% (80/230) and 38.2% (71/186) in Asella, Adama and Semara, and BP threshold was reached in 48.5% (95/196), 27.8% (64/230) and 36.0% (67/186), respectively. Remarkably, in Asella, at high altitude, the median RR in the analyzed healthy population reached the qSOFA score threshold (Fig 3). As presented in Table 3, across all sites 16.7% (102/612) of participants scored 2 points in the qSOFA score (RR≥22 and BP_sys_≤100 mmHg). In particular, the qSOFA score reached 2 points in 28.1% (55/196) of participants in Asella, in 8.3% (19/230) of participants in Adama and 15.1% (28/186) of participants in Semara (see Fig 4). Notably, the distribution of the qSOFA score in Asella was significantly different from Adama and Semara (p<0.001, Table 3 and Fig 4). Also, when adjusting for the covariates age, sex and BMI in the multiple ordinal regression model, Asella as area of residency was significantly associated with an elevated qSOFA score compared to the location Adama (aOR 3.26 [2.20–4.86], p<0.001, Table 4), whereas no difference between Semara and Adama was observed. Furthermore, male gender was significantly associated with a lower qSOFA score (p = 0.004).

**Table 2:** Physiological parameters at the different study sites.

	Asella, 2400 m a.s.l. (A)	Adama, 1620 m a.s.l. (B)	Semara, 400 m a.s.l. (C)	p-value (group differences)	p-value (Single group comparisons)		
					A|B	A|C	B|C
Temperature in °C, median (IQR)	36.7 (36.5–36.9)	36.5 (36.3–36.8)	36.6 (36.1–37.0)	<0.001	<0.001	0.009	0.555
Heart rate in /min, median (IQR)	80 (72–90)	81 (73–90)	83 (74–91)	0.233	0.90	0.21	0.21
Systolic blood pressure in mmHg, median (IQR)	110 (100–113)	110 (100–120)	110 (100–120)	<0.001	<0.001	0.004	0.017
02 saturation in %, median (IQR)	96 (95–97)	97 (96–98)	98 (98–99)	<0.001	<0.001	<0.001	<0.001
Respiratory rate in /min, median (IQR)	22 (20–24)	20 (18–22)	21 (19–23)	<0.001	<0.001	<0.001	0.022
Body mass index in kg/m^2^, median (IQR)	21.7 (19.4–23.6)	24.05 (20.8–26.7)	20.6 (18.6–23.1)	<0.001	<0.001	0.009	<0.001

BMI: Body Mass Index

**Table 3:** Proportions of the qSOFA at the different study sites.

qSOFA-score, n (%)	Total (n = 612)	Asella (n = 196) (A)	Adama (n = 230) (B)	Semara (n = 186) (C)	p-value (group differences)	p-value (Single group comparisons)		
						A|B	A|C	B|C
0	218 (35.6)	37 (18.9)	105 (45.7)	76 (40.9)	<0.001	<0.001	<0.001	0.089
1	292 (47.7)	104 (53.1)	106 (46.1)	82 (44.1)				
2	102 (16.7)	55 (28.1)	19 (8.3)	28 (15.1)				

qSOFA: Quick Sequential Organ Failure Assessment

**Table 4:** Factors associated with the qSOFA at the different study sites.

	qSOFA		
Covariates	aOR	95% CI	p-value
Age in years	0.99	(0.98–1.01)	0.247
Female	1		
Male	0.61	(0.43–0.86)	**0.004**
BMI in kg/m^2^	0.98	(0.94–1.02)	0.295
Adama	1		
Asella	3.26	(2.20–4.86)	**<0.001**
Samara	1.32	(0.89–1.96)	0.171

qSOFA: Quick Sequential Organ Failure Assessment, aOR: adjusted Odds Ratios, CI: Confidence Interval, BMI: Body Mass Index

## Discussion

Within the cohort of healthy adult volunteers, the high proportion of individuals reaching the cutoff for a positive qSOFA score was surprising. The qSOFA score has been developed as an easy-to-use bedside score in order to quickly identify individuals at risk of a poor outcome among patients with an infection [19]. In previous studies, positive qSOFA criteria showed a similar prognostic significance compared with more complex tools as the SOFA, MEDS (Mortality in Emergency Department Sepsis) or APACHE II (Acute Physiology And Chronic Health Evaluation II) scores [20]. The specificity to predict mortality among patients with an infection of the qSOFA score in sub-Saharan African cohorts was reported to be 82% (95% Confidence Interval [CI] 76–88) and 81% (95% CI 78–85) while sensitivity was much lower (55% [95% CI 23–83] and 44% [95% CI 33–55], respectively) [5, 7].

All study participants were alert pedestrians, were not apparently mentally altered, as assessed during study procedures, and did not report to suffer from chronic illness. Thus, all patients were considered to have an unimpaired GCS of 15. The RR was significantly higher and the SpO_2_ significantly lower in Asella, the site situated at the highest altitude a.s.l., whereas there was no significant difference between the sites at lower altitudes. Although the rate of positive qSOFA was also remarkably high at those sites, we postulate that the high altitude of 2400 m a.s.l. might contribute to the high rate of unspecific positive qSOFA score values. As it has been suggested by other authors [5], an adaption of existing scores to various settings might be necessary to improve the performance. Alternatively, to improve the impaired performance of clinical scores at different altitudes, a constant conversion factor could possibly be derived from future cohort analyses.

The finding of a positive qSOFA score in the normal population was mostly common in Asella, located at 2400 m a.s.l. It has been shown elsewhere, that the qSOFA can be a reliable predictor of mortality, also in resource-limited settings [6, 21, 22] but perhaps not at extreme elevations. Our hitherto deviating results can only partly be explained by comparatively high altitudes in Ethiopia, since also at lower altitude, in Semara at 400 m a.s.l., a positive qSOFA was frequently found and was present in 15.1% of the local population. However, the median RR in the normal healthy population at Asella reached the threshold of the qSOFA score and even though the median BP_sys_ was 110 mmHg at all three sites, there was a significant difference in the IQR between the three sites, being lowest in participants from Asella. These findings explain the high rate of healthy individuals reaching a positive qSOFA. On the other hand, body temperature and HR, both parameters not included in the qSOFA score, showed no or hardly any differences between the study sites at different altitudes.

A limited specificity of the score in the studied population has already been described in previous studies conducted in countries with limited resources in Sub-Saharan Africa [5, 10, 23, 24] and may be partially explained by deviations of the standard values of different vital signs like RR or BP_sys_ although optimal thresholds remain uncertain. Our results support previous findings questioning the accurate applicability of the qSOFA not only in resource-limited settings, but also in more developed settings [8, 25, 26]. With a high proportion of positive qSOFA criteria in a normal population, the score fails to serve as specific tool for the identification of septic patients at risk for adverse outcomes.

Our findings might be limited due to the fact that our data was recorded as a single cross-sectional assessment and do not reflect physiological variation of the parameters within individuals. Nevertheless, also vital parameters used to calculate sepsis scores are usually assessed once at a certain time point in clinical settings. The analysis of multiple individuals within our cohort reduces the risk for selection bias. Since all study procedures were performed using volunteers, the results could be influenced by volunteer bias. However, since the sampling methods did not differ between the study sites, this bias cannot explain the apparent differences between the different study groups. No extrinsic motivation in form of any compensation was offered for participants. The conducted post-hoc power analysis indicated a sufficiently large group size to test the study objective.

Participants reporting any form of chronic disease were excluded from further analysis to rule out the possibility of changes in vital signs caused by illness. However, Patel et al. were able to show that self-reporting leads to limitations in the reliability of chronic disease detection [27]. Thus, among the participants classified as healthy in this study, there may have been individuals potentially suffering from a chronic disease. Since this limitation applies equally to all study centers, no distortion of the study results in the comparison of the study centers is to be expected.

The European Society of Cardiology, the American Heart Association and others suggest having a patient rest for 5 minutes before measuring BP [16, 17, 28]. This approach was followed during our study. However, there are other data which indicate that a longer resting time of 10 or even 25 minutes might be necessary for reliable stabilization of BP [29, 30]. To circumvent a resulting error, no volunteers apparently exhausted by physical activity were included and the same procedure was followed at all study sites.

Also, the differences among demographic parameters such as age, gender and BMI between the study sites have to be considered as possible limitation of the study, but for this very reason the ordinal regression model was adjusted using these parameters and it was shown that Asella as place of residence is associated with a higher probability of a false positive qSOFA score, regardless of age, gender and BMI. Possibly, this finding could also be confounded by different socio-economic and environmental conditions (e.g. climate, air pollution) at the three study sites with Adama as a metropolis, Asella as a major district town and Semara as rather remote city. The varying BMI at the different study populations could reflect the respective economic strength and might be interpreted as an indication of different lifestyles of the population at the study sites. However, this assumption is based on personal observations and reliable data to support this hypothesis are insufficient.

## Conclusion

Our study indicates that the applicability of the qSOFA score or other clinical scores based on examination of the vital signs BP and RR may be adversely affected by shifts in the range of normal values of the vital signs, e.g. as an adaptation mechanism for altitude. As it has previously been suggested by the *international Sepsis-3 Task force*, the qSOFA needs further investigation and validation especially in resource-limited health care settings [19]. High altitude might potentially be a relevant factor, since large populations of around 389 million people live in altitudes above 1.500 m, especially in Mexico, South America, the South-Central Asian Highlands, and Eastern Africa (Kenya, Ethiopia) [31]. Adaption of scores based on physiological parameters, as the qSOFA, according to local variances could improve the performance of these scores.

## Supporting information

S1 Dataset Minimal dataset. (XLSX)

## References

[1] Barfod C, Lauritzen MMP, Danker JK, et al Abnormal vital signs are strong predictors for intensive care unit admission and in-hospital mortality in adults triaged in the emergency department—a prospective cohort study. *Scand J Trauma Resusc Emerg Med* 2012; 20: 28 10.1186/1757-7241-20-28 22490208 PMC3384463

[2] Farrohknia N, Castrén M, Ehrenberg A, et al Emergency department triage scales and their components: a systematic review of the scientific evidence. *Scand J Trauma Resusc Emerg Med* 2011; 19: 42 10.1186/1757-7241-19-42 21718476 PMC3150303

[3] Freund Y, Lemachatti N, Krastinova E, et al Prognostic Accuracy of Sepsis-3 Criteria for In-Hospital Mortality Among Patients With Suspected Infection Presenting to the Emergency Department. *JAMA* 2017; 317: 301–308. 10.1001/jama.2016.20329 28114554

[4] Rudd KE, Seymour CW, Aluisio AR, et al Association of the Quick Sequential (Sepsis-Related) Organ Failure Assessment (qSOFA) Score With Excess Hospital Mortality in Adults With Suspected Infection in Low- and Middle-Income Countries. *JAMA* 2018; 319: 2202–2211. 10.1001/jama.2018.6229 29800114 PMC6134436

[5] Schmedding M, Adegbite BR, Gould S, et al A Prospective Comparison of Quick Sequential Organ Failure Assessment, Systemic Inflammatory Response Syndrome Criteria, Universal Vital Assessment, and Modified Early Warning Score to Predict Mortality in Patients with Suspected Infection in Gabon. *Am J Trop Med Hyg* 2019; 100: 202–208. 10.4269/ajtmh.18-0577 30479248 PMC6335900

[6] Huson MAM, Katete C, Chunda L, et al Application of the qSOFA score to predict mortality in patients with suspected infection in a resource-limited setting in Malawi. *Infection* 2017; 45: 893–896. 10.1007/s15010-017-1057-5 28786004 PMC5696439

[7] Aluisio AR, Garbern S, Wiskel T, et al Mortality outcomes based on ED qSOFA score and HIV status in a developing low income country. *Am J Emerg Med* 2018; 36: 2010–2019. 10.1016/j.ajem.2018.03.014 29576257 PMC6886365

[8] Churpek MM, Snyder A, Han X, et al Quick Sepsis-related Organ Failure Assessment, Systemic Inflammatory Response Syndrome, and Early Warning Scores for Detecting Clinical Deterioration in Infected Patients outside the Intensive Care Unit. *Am J Respir Crit Care Med* 2017; 195: 906–911. 10.1164/rccm.201604-0854OC 27649072 PMC5387705

[9] Zhang HL, Crump JA, Maro VP, et al Predicting Mortality for Adolescent and Adult Patients with Fever in Resource-Limited Settings. *Am J Trop Med Hyg* 2018; 99: 1246–1254. 10.4269/ajtmh.17-0682 30226134 PMC6221229

[10] Niyongombwa I, Sibomana I, Karenzi ID, et al Kigali Surgical Sepsis (KiSS) Score: A New Tool to Predict Outcomes in Surgical Patients with Sepsis in Low- and Middle-Income Settings. *World J Surg* 2020; 44: 3651–3657. 10.1007/s00268-020-05708-7 32700110

[11] Caffrey D, Miranda J, Gilman RH, et al A cross-sectional study of differences in 6-min walk distance in healthy adults residing at high altitude versus sea level. *Extreme Physiol Med* 2014; 3: 3 10.1186/2046-7648-3-3 24484777 PMC3909455

[12] Aryal N, Weatherall M, Bhatta YKD, et al Blood Pressure and Hypertension in Adults Permanently Living at High Altitude: A Systematic Review and Meta-Analysis. *High Alt Med Biol* 2016; 17: 185–193. 10.1089/ham.2015.0118 27575245

[13] Toselli S, Tarazona-Santos E, Pettener D. Body size, composition, and blood pressure of high-altitude Quechua from the Peruvian Central Andes (Huancavelica, 3,680 m). *Am J Hum Biol* 2001; 13: 539–547. 10.1002/ajhb.1086 11400225

[14] Mingji C, Onakpoya IJ, Perera R, et al Relationship between altitude and the prevalence of hypertension in Tibet: a systematic review. *Heart* 2015; 101: 1054–1060. 10.1136/heartjnl-2014-307158 25953970 PMC4484261

[15] Sizlan A, Ogur R, Ozer M, et al Blood pressure changes in young male subjects exposed to a median altitude. *Clin Auton Res* 2008; 18: 84–89. 10.1007/s10286-008-0459-y 18363033

[16] Williams B, Mancia G, Spiering W, et al 2018 Practice Guidelines for the management of arterial hypertension of the European Society of Hypertension and the European Society of Cardiology: ESH/ESC Task Force for the Management of Arterial Hypertension. *J Hypertens* 2018; 36: 2284–2309. 10.1097/HJH.0000000000001961 30379783

[17] Whelton PK, Carey RM, Aronow WS, et al 2017 ACC/AHA/AAPA/ABC/ACPM/AGS/APhA/ASH/ASPC/NMA/PCNA Guideline for the Prevention, Detection, Evaluation, and Management of High Blood Pressure in Adults: Executive Summary: A Report of the American College of Cardiology/American Heart Association Task Force on Clinical Practice Guidelines. *Hypertens Dallas Tex 1979* 2018; 71: 1269–1324. 10.1161/HYP.0000000000000066 29133354

[18] Cohen J. *Statistical power analysis for the behavioral sciences*. 2nd ed Hillsdale, N.J: L. Erlbaum Associates, 1988.

[19] Singer M, Deutschman CS, Seymour CW, et al The Third International Consensus Definitions for Sepsis and Septic Shock (Sepsis-3). *JAMA* 2016; 315: 801–810. 10.1001/jama.2016.0287 26903338 PMC4968574

[20] Wang J-Y, Chen Y-X, Guo S-B, et al Predictive performance of quick Sepsis-related Organ Failure Assessment for mortality and ICU admission in patients with infection at the ED. *Am J Emerg Med* 2016; 34: 1788–1793. 10.1016/j.ajem.2016.06.015 27321936

[21] Fernandes S, Wyawahare M. Utility of quick sepsis-related organ failure assessment (qSOFA) score to predict outcomes in out-of-ICU patients with suspected infections. *J Fam Med Prim Care* 2020; 9: 3251 10.4103/jfmpc.jfmpc_150_20 33102279 PMC7567244

[22] Kayambankadzanja RK, Schell CO, Namboya F, et al The Prevalence and Outcomes of Sepsis in Adult Patients in Two Hospitals in Malawi. *Am J Trop Med Hyg* 2020; 102: 896–901. 10.4269/ajtmh.19-0320 32043446 PMC7124904

[23] Moore CC, Hazard R, Saulters KJ, et al Derivation and validation of a universal vital assessment (UVA) score: a tool for predicting mortality in adult hospitalised patients in sub-Saharan Africa. *BMJ Glob Health* 2017; 2: e000344 10.1136/bmjgh-2017-000344 29082001 PMC5656117

[24] Beane A, De Silva P, Munasinghe S, et al Comparison of quick sequential organ failure assessment and modified systemic inflammatory response syndrome criteria in a lower middle income setting. *J Acute Med* 2017; 7: 141–148. 10.6705/j.jacme.2017.0704.002 32995188 PMC7517879

[25] Williams JM, Greenslade JH, McKenzie JV, et al Systemic Inflammatory Response Syndrome, Quick Sequential Organ Function Assessment, and Organ Dysfunction: Insights From a Prospective Database of ED Patients With Infection. *Chest* 2017; 151: 586–596. 10.1016/j.chest.2016.10.057 27876592

[26] Garbero R de F, Simões AA, Martins GA, et al SOFA and qSOFA at admission to the emergency department: Diagnostic sensitivity and relation with prognosis in patients with suspected infection. *Turk J Emerg Med* 2019; 19: 106–110. 10.1016/j.tjem.2019.05.002 31321343 PMC6612625

[27] Patel S, Ram F, Patel SK, et al Association of behavioral risk factors with self-reported and symptom or measured chronic diseases among adult population (18–69 years) in India: evidence from SAGE study. *BMC Public Health* 2019; 19: 560 10.1186/s12889-019-6953-4 31088447 PMC6518500

[28] Whitworth JA, World Health Organization, International Society of Hypertension Writing Group. 2003 World Health Organization (WHO)/International Society of Hypertension (ISH) statement on management of hypertension. *J Hypertens* 2003; 21: 1983–1992. 10.1097/00004872-200311000-00002 14597836

[29] Mahe G, Comets E, Nouni A, et al A minimal resting time of 25 min is needed before measuring stabilized blood pressure in subjects addressed for vascular investigations. Sci Rep; 7 Epub ahead of print 10 October 2017. 10.1038/s41598-017-12775-9 29018246 PMC5635024

[30] Sala C, Santin E, Rescaldani M, et al How long shall the patient rest before clinic blood pressure measurement? *Am J Hypertens* 2006; 19: 713–717. 10.1016/j.amjhyper.2005.08.021 16814126

[31] Cohen JE, Small C. Hypsographic demography: The distribution of human population by altitude. *Proc Natl Acad Sci* 1998; 95: 14009–14014. 10.1073/pnas.95.24.14009 9826643 PMC24316

